# The potential of yeast culture for alleviating enterotoxigenic *Escherichia coli*-induced diarrhea in neonatal livestock: Insights from intestinal barrier mechanisms

**DOI:** 10.1016/j.aninu.2025.12.004

**Published:** 2026-03-05

**Authors:** Shuai Wang, Pengxiang Bai, Hui Chen, Lan Yang, Dacheng Liu

**Affiliations:** a College of Veterinary Medicine, Inner Mongolia Agricultural University, Hohhot 010018, Inner Mongolia, China; b National Dairy Technology Innovation Center, Hohhot 010105, Inner Mongolia, China

**Keywords:** Yeast culture, Enterotoxigenic *Escherichia coli*, Diarrhea, Livestock, Intestinal barrier

## Abstract

Enterotoxigenic *Escherichia coli* (ETEC) remains a leading cause of neonatal diarrhea in piglets, calves, and lambs, yet antibiotic curtailment demands nutrition-based solutions. This review takes a novel barrier-centric perspective to evaluate how yeast culture mitigates neonatal livestock diarrhea caused by ETEC. Unlike previous probiotic or prebiotic reviews, this review focuses specifically on multifaceted components of yeast culture and map their protective effects on each intestinal barrier layer. Across challenge and field studies, yeast culture supplementation reduced neonatal diarrhea incidence relatively by up to 40% to 60% and improved weight gain, highlighting its practical efficacy. Consistent biological correlates include preserved tight junction protein expression and epithelial permeability, thicker mucus and higher goblet-cell count, increased mucosal secretory immunoglobulin A (sIgA) with tempered cytokine responses, and microbiota shifts toward colonization enhanced colonization resistance and short-chain fatty acid production, thereby collectively fortifying the gut against ETEC adhesion and toxin-induced damage. Despite these promising outcomes, challenges remain in optimizing yeast formulation and dosage, and further research is needed to address gaps in species-specific application and long-term efficacy. Overall, yeast culture may provide a multi-target, antibiotic-free strategy to reduce ETEC-associated diarrhea by restoring barrier function and microbiota homeostasis.

## 1 Introduction

Enterotoxigenic *Escherichia coli* (ETEC) is a primary causative agent of diarrhea in neonatal livestock, affecting economically important species such as pigs, cattle, and sheep (Dubreuil et al., 2016). Enterotoxigenic *E*. *coli* compromises intestinal barrier integrity through the secretion of two major enterotoxins: heat-labile toxin (LT) and heat-stable toxin (ST) (Higginson et al., 2022). These toxins bind to receptors on the intestinal epithelial cell surface and activate intracellular signaling cascades, ultimately disrupting tight junctions (TJs) and impairing barrier function (Kim et al., 2022). Barrier dysfunction not only leads to excessive secretion of electrolytes and water, but also facilitates bacterial translocation, resulting in severe diarrhea (Su et al., 2022). Additionally, ETEC infection suppresses the host immune responses and triggers inflammatory cascades that exacerbate mucosal injury and impair intestinal function, thereby potentially hindering growth and increasing mortality rates in young animals (Kim et al., 2022). Thus, elucidating the pathogenic mechanisms of ETEC associated with neonatal diarrhea and developing effective countermeasures remain pressing challenges in animal production.

Historically, antibiotics have been widely employed to manage ETEC-induced diarrhea. However, their overuse has contributed to escalating antimicrobial resistance and raised concerns regarding environmental and public health impacts (Laird et al., 2021). For instance, China's 2020 ban on in-feed antibiotic growth promoters and the EU's 2022 ban on prophylactic zinc oxide in piglets have created a critical need for alternative solutions (Liu et al., 2023; Nielsen et al., 2025). Despite rising antimicrobial resistance and industry demand for drug-free strategies, neonatal calf diarrhea prevalence still approaches 27% in China (Qu et al., 2023), underscoring the need for new interventions. Yeast culture, a natural feed additive, has garnered attention due to its beneficial effects on gut health. Yeast and its fermentation products have been shown to modulate gut microbiota, enhance mucosal immunity, and reinforce intestinal barrier integrity (Maturana et al., 2023). Evidence suggests that yeast culture reduces the incidence and mortality of diarrhea in neonates by promoting probiotic growth, strengthening epithelial TJs, and activating immune cells (Liu et al., 2024). Components such as cell-wall polysaccharides and metabolic products exert anti-inflammatory and immunomodulatory effects, inhibit pathogen adhesion, and stimulate host defenses, thereby preserving barrier function (Patterson et al., 2023). Yeast culture combines prebiotic (cell wall polysaccharides) and postbiotic (metabolic products) features within a standardizable feed ingredient, positioning it as a pragmatic alternative to antibiotics (Maturana et al., 2023). Unlike single ingredients, yeast culture provides a broader set of component–receptor interactions and metabolite-level effects that converge on barrier repair, which may offer a more holistic defense against ETEC than single-component supplements.

Prior reviews have examined broad probiotics and fibers, but none have synthesized yeast culture evidence through an intestinal barrier framework. This review addresses that gap by focusing on how yeast culture and its components strengthen each layer of the gut barrier against ETEC. This review also highlights unresolved questions (e.g., inconsistent results across studies and optimal dosing) and propose future research directions. In summary, this barrier-centric review of yeast culture is timely and valuable, as it aligns with antibiotic reduction strategies and the growing need for sustainable control of neonatal diarrhea.

## 2 Pathogenic mechanism of ETEC

Enterotoxigenic *E.* *coli* pathogenesis involves two key steps: initial colonization of the small intestinal mucosa via adhesion factors, followed by secretion of enterotoxins that impair intestinal barrier function. This leads to electrolyte imbalance, fluid loss, and severe diarrhea (Zhang et al., 2022). A thorough understanding of how ETEC disrupts intestinal barrier integrity through the combined actions of adhesion factors and enterotoxins is crucial for the development of effective preventive and therapeutic strategies. Because this review centers on yeast culture as an antibiotic-free strategy, this review summarizes ETEC pathogenesis only to the extent that clarifies where yeast culture can intervene.

### 2.1 Adhesion factors

The pathogenic process of ETEC begins with adhesion, which is essential for bacterial colonization. Adhesion factors, or colonization factors, are specialized surface structures on ETEC that bind to specific receptors on the host intestinal epithelium, facilitating bacterial attachment (Von Mentzer and Svennerholm, 2024). Common adhesion types include F4, F5, F18, and F41. In neonatal animals such as piglets, calves, and lambs, intestinal epithelial cells express specific receptors for these fimbriae, enabling effective colonization (Dubreuil et al., 2016). Once established, ETEC proliferates within the intestinal lumen and secretes enterotoxins that disrupt host intestinal function. Thus, ETEC's adhesion phase not only initiates infection, but also actively weakens the intestinal barrier by eroding the mucus layer and evading local immunity. These insights underscore the importance of strategies that disrupt bacterial attachment and preserve the mucus barrier. For instance, yeast culture is rich in mannan components, which can act as decoy receptors for adhesins and promote aggregation/adsorption of pathogenic *E. coli*, potentially lowering effective adhesion pressure at the mucus–epithelium interface (Faustino et al., 2021). These actions create a window for barrier function to recover under challenge.

### 2.2 Enterotoxins

Following successful colonization, ETEC releases ST and LT that trigger fluid secretion via distinct but convergent pathways in the small intestine. Heat-stable toxin activates epithelial guanylate cyclase-C (GC-C), elevating cyclic guanosine monophosphate (cGMP) to open chloride channels, producing secretory diarrhea (Barros et al., 2025). Heat-labile toxin binds the ganglioside GM1 to deliver its A subunit, which activates adenylate cyclase (AC), raises cyclic adenosine monophosphate (cAMP), and stimulates protein kinase A-dependent chloride secretion, causing secretory diarrhea (Park et al., 2023). These enterotoxins disrupt electrolyte and water homeostasis, resulting in secretory diarrhea, dehydration, mucosal barrier disruption, and further impairment of intestinal barrier function (Zhang et al., 2022). A crucial implication is that fortifying the barrier may mitigate toxin impact: if TJs remain intact and the mucus layer is thick, ST and LT have reduced access to their receptors, and the downstream cyclic guanosine monophosphate/cyclic adenosine monophosphate overactivation is blunted. For instance, by reinforcing TJs and thickening mucus, yeast culture components may reduce access of ST/LT to the epithelium and dampen downstream cGMP/cAMP-driven secretion and inflammation (Luo et al., 2024). Additionally, β-glucans and mannans modulate innate signaling that may shape the early response to toxins (Nam et al., 2024). In the context of this review, these observations suggest that yeast culture, by reinforcing barrier structures, may counteract the pathological cycle of ETEC toxins.

In summary, each step in ETEC's pathogenic cycle is matched by a defensive mechanism from yeast culture. ETEC fimbriae attempt to colonize, whereas yeast mannans block adhesion. Enterotoxigenic *E*. *coli* toxins disrupt fluid balance and barrier integrity, whereas yeast components fortify TJs, enhance mucus secretion, and modulate host responses to maintain homeostasis.

## 3 Intestinal barrier: Structure and function

The intestinal barrier is a critical defense system that protects the host from pathogenic invasion while ensuring proper nutrient and fluid absorption (Patra et al., 2019). When functioning normally, it maintains internal homeostasis and prevents infections. However, barrier impairment allows pathogens to penetrate, increasing the risk of intestinal and systemic diseases (Rogers et al., 2023). In young animals, where the barrier is not fully mature, ETEC infection can cause significant disruption, leading to diarrhea and immune imbalance (Yu et al., 2021c). This review examines how each layer of the intestinal barrier is compromised by ETEC and then explicitly link those injuries to the protective actions of yeast culture.

### 3.1 Mechanical barrier

The mechanical barrier is primarily formed by intestinal epithelial cells and their intercellular TJs (Higashi et al., 2024). During ETEC infection, the STb enterotoxin binds to specific epithelial receptors, such as sulfatide, and is internalized, triggering cytoskeletal rearrangement of F-actin and subsequent redistribution or degradation of TJ proteins. This process compromises epithelial integrity, increasing intestinal permeability (Dubreuil, 2017). In vitro studies using T84 epithelial cells have shown that STb significantly reduces transepithelial electrical resistance and disrupts the localization of zonula occludens-1, claudin-1, and occludin within 24 h (Serek and Oleksy-Wawrzyniak, 2021). In neonatal piglets, weaning stress further weakens the gut barrier, and ETEC infection leads to marked downregulation of TJs protein expression, exacerbating barrier dysfunction. Such damage facilitates bacterial translocation and toxin entry into systemic circulation, promoting inflammation, diarrhea, and dehydration (Wang and Ji, 2019). The above evidence underscores that TJs disruption is a central driver of ETEC-induced gut leakage and diarrhea. It follows that preserving TJs integrity or accelerating its restoration after insult is crucial for mitigating disease severity. Indeed, these TJs lesions create a therapeutic window in which yeast culture restores TJs protein localization and reduces paracellular flux in vivo and in vitro. In weaned pigs and IPEC-J2 models, yeast culture upregulates zonula occludens-1/claudin-1 and improves transepithelial electrical resistance via pattern-recognition receptor (PRR) nucleotide binding oligomerization domain containing 1/nuclear factor kappa-B p65 pathways, thereby accelerating TJs recovery (Wang et al., 2020). Recent study using mannan oligosaccharides (MOS)-selenium also reported that dietary MOS-selenium supplementation significantly decreased intestinal barrier permeability and alleviated ETEC-induced intestinal mucosal barrier injury (Zha et al., 2023). While multiple studies have shown that yeast can improve transepithelial resistance and TJ protein levels, it remains unclear how different yeast formulations compare in their efficacy, which represents an area for further study.

### 3.2 Chemical barrier

The chemical barrier of the intestine is primarily composed of the mucus layer covering the mucosal surface and antimicrobial substances secreted by epithelial cells (Gieryńska et al., 2022). However, during ETEC infection, the chemical barrier is significantly compromised. On one hand, ETEC utilizes adhesins (e.g., F18) and flagellar motility to penetrate the mucus layer and reach the epithelial surface (Sauvaitre et al., 2022). On the other hand, certain ETEC strains secrete mucin (MUC)-degrading enzymes such as secreted and surface-associated lipoprotein E, which break down key MUCs like MUC2 and MUC3, leading to local thinning or disruption of the mucus layer. This degradation facilitates pathogen access to epithelial cells, promoting colonization and intensifying infection (Luo et al., 2014). Moreover, ETEC toxins alter the physicochemical properties of mucus by downregulating MUC-related gene expression in goblet cells, thereby reducing mucus production (Cai et al., 2020). In summary, ETEC actively erodes the mucus barrier, which is a linchpin in preventing infection. This highlights an important defensive strategy: maintaining or enhancing mucus production may counteract ETEC's tactics. A thicker, continuously replenished mucus layer makes it much harder for ETEC to reach and attach to epithelial cells. Therefore, interventions that stimulate goblet cells or otherwise fortify the mucus are likely to blunt ETEC colonization. Recent mechanistic work pinpoints ETEC autotransporter A–MUC2 cleavage as a determinant of epithelial access, arguing for adjunct strategies that preserve MUC2 (e.g., yeast culture-supported mucus biology) (Sheikh et al., 2022). Dietary yeast-based MOS enhances goblet cell function and mucus architecture (Leclercq et al., 2020), while *Saccharomyces cerevisiae*-derived postbiotic alter the gut environment to optimize luminal chemistry that supports mucus viscoelasticity (Duysburgh et al., 2024). Mucus-ETEC interaction is now recognized as a central pathogenic node, strengthening the rationale to target the MUC2 erosion with yeast culture components. Yeast components clearly boost mucus, but whether this translates to sustained mucus protection under continuous field exposure is not fully established, warranting longer-term in vivo studies.

### 3.3 Immune barrier

The intestinal immune barrier constitutes a critical component of the gut's defense system. It is composed of mucosa-associated lymphoid tissue, intestinal lymph nodes, Peyer's patches, and sIgA (Niewiem and Grzybowska-Chlebowczyk, 2022). However, ETEC infection can disrupt this immune balance. Enterotoxigenic *E.* *coli* adheres to epithelial cells via adhesins, inducing immune activation and promoting the secretion of pro-inflammatory cytokines such as interleukin-1β, interleukin-6, and tumor necrosis factor-α. These cytokines recruit neutrophils and macrophages to the mucosal site, triggering inflammation (Osorio, 2020). Studies have shown that F18^+^ ETEC infection in piglets significantly elevates interleukin-6 and interleukin-8 levels in the small intestinal mucosa, accompanied by pronounced inflammation and tissue injury. Excessive inflammation damages epithelial cells and compromises barrier integrity (Kim et al., 2022). In addition, the LT toxin secreted by ETEC acts as an immunological adjuvant, inducing polyclonal B cell activation and depleting immune resources, which may ultimately result in immunosuppression (Menge, 2020). Prolonged diarrhea exacerbates immune dysfunction by causing the loss of protective molecules such as sIgA, further weakening mucosal immunity. This immune imbalance increases susceptibility to infection, perpetuating a vicious cycle (Tao and Lang, 2024). Breaking this cycle requires restoring immune homeostasis and boosting protective immunity while preventing excessive inflammatory damage. In practical terms, approaches that enhance sIgA secretion and temper the overproduction of cytokines may fortify the immune barrier against ETEC. For instance, yeast culture, through its diverse bioactive components, including β-glucans and MOS, can modulate mucosal immune responses through various pathways, enhancing sIgA levels and inhibiting the release of inflammatory cytokines. *S.* *cerevisiae* β-glucans signal through Dectin-1 and can induce trained innate immunity (Vuscan et al., 2024), elevating mucosal sIgA and rebalancing cytokines (Ikewaki et al., 2021). Mannan oligosaccharides can tune Tolllike receptors-dependent cytokine output, collectively restraining toxin-driven inflammation while preserving mucosal defense (Rehman et al., 2021). These effects improve the local intestinal immune environment. A remaining question is which immune pathways are most crucial; for example, is the sIgA increase or the cytokine suppression the dominant factor in protection? Further mechanistic immunology work with yeast components could clarify this.

### 3.4 Microbial barrier

The intestinal microbial barrier serves as a vital ecological defense against pathogenic invasion, including ETEC. It protects host health through niche competition, production of antimicrobial metabolites, and activation of local immune responses (Rooks and Garrett, 2016). However, ETEC infection disrupts this microbial equilibrium. Studies have shown a significant reduction in the abundance of beneficial taxa such as *Lactobacillus* and *Prevotella* in the small and large intestines of diarrheic piglets, accompanied by an increase in pathogenic *E. coli* (Gryaznova et al., 2022). Using 16S rRNA gene sequencing, Bin et al. (2018) demonstrated that ETEC-induced diarrhea leads to a marked decline in microbial diversity, with a reduced abundance of *Bacteroidetes* and elevated levels of pathogenic *E. coli* and *Shigella* in feces. These shifts reflect a breakdown of the microbial barrier, facilitating rapid colonization and spread of pathogens. Notably, fecal microbiota transplantation from ETEC-infected piglets into germ-free recipients reproduced diarrheal symptoms, underscoring the microbiota's causal role in disease pathogenesis (Mooyottu et al., 2025). These findings make clear that dysbiosis is a major contributor to ETEC pathology. Therefore, restoring a healthy microbial community is a pivotal therapeutic angle. Strategies that boost beneficial short-chain fatty acid (SCFA)-producing bacteria and curtail pathogen overgrowth can re-establish the gut's microbial barrier. This is precisely the approach of certain prebiotic or postbiotic interventions. For example, yeast culture, as a natural microbiome regulator, plays a significant role in maintaining microbial diversity, promoting the proliferation of beneficial bacteria, and modulating metabolic products. Yeast culture modulates community structure toward Lactobacillaceae and Bifidobacteriaceae, and increases SCFAs, strengthening colonization resistance against ETEC (Cherrington et al., 2025). While yeast-driven microbiome improvements are documented, the causal links between specific microbiome changes and improved disease resistance need further elucidation.

**Fig. 1:**
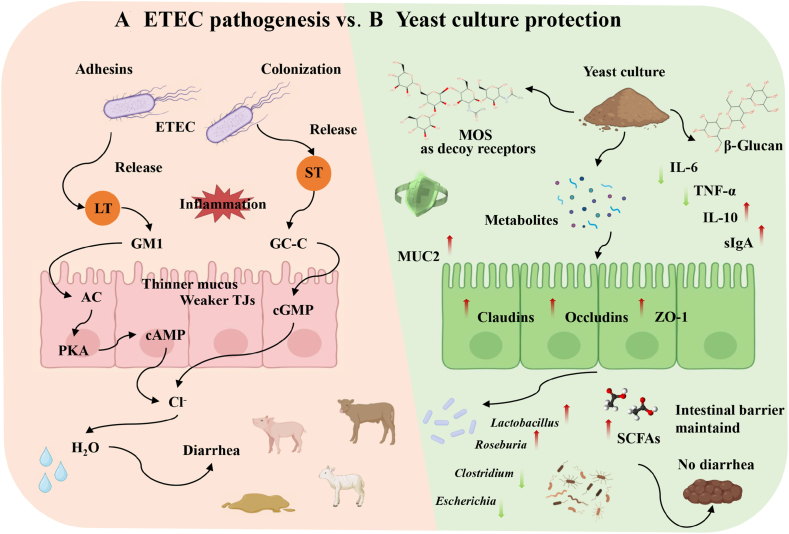
Summary of enterotoxigenic *Escherichia coli* (ETEC) pathogenesis and yeast culture's protective actions. (A) In ETEC infection, adhesins enable bacterial adhesion to the intestinal epithelium, leading to colonization and enterotoxin (LT and ST) release. Toxins disrupt TJs and stimulate electrolyte secretion, causing barrier damage (leaky epithelium, mucus degradation, and inflammation) and resulting in severe diarrhea. (B) Yeast culture delivers a multi-component intervention: mannans act as decoy receptors that block ETEC adhesion to the gut lining; β-glucans modulate immunity (enhancing sIgA/IL-10, reducing IL-6/TNF-α) to dampen inflammation; and fermentation-derived metabolites (organic acids and SCFAs) strengthen the barrier by preserving TJs, stimulating mucus production, and maintaining a healthy gut microbiota. These mechanisms may collectively maintain intestinal integrity, preventing fluid loss and supporting normal absorption. LT = heat-labile toxin; ST = heat-stable toxin; AC = adenylate cyclase; PKA = protein kinase A; cAMP = cyclic adenosine monophosphate; cGMP = cyclic guanosine monophosphate; GC-C = guanylate cyclase-C; TJs = tight junctions; MOS = mannan oligosaccharides; MUC2 = mucin 2; ZO-1 = zonula occludens-1; SCFAs = short-chain fatty acids; IL-6 = interleukin-6; IL-10 = interleukin-10; TNF-α = tumor necrosis factor-α; sIgA = secretory immunoglobulin A. Red arrow: increase; green arrow: decrease.

In summary, ETEC impairs all four layers of the intestinal barrier. These impairments not only facilitate ETEC colonization and invasion, but also exacerbate mucosal inflammation and diarrheal severity (Zhang et al., 2022). Therefore, targeting these pathogenic pathways—particularly by restoring barrier integrity—represents a promising strategy for mitigating ETEC-induced diarrhea. Yeast culture, through its rich functional components and metabolic products, comprehensively protects the intestinal barriers at various levels. This multi-targeted synergistic action makes yeast culture a promising tool for preventing and alleviating ETEC-induced diarrhea in young livestock (Fig. 1). It is also notable that not all supplements are equal in this regard; differences in yeast product composition lead to varying degrees of barrier recovery, highlighting the need to identify the most effective formulations.

## 4 Potential mechanism of yeast culture

Produced by fermenting *S.* *cerevisiae* and then drying together with its medium, yeast culture contains both the structural cell-wall fractions and an array of bioactive metabolites (Pang et al., 2022). Yeast culture has gained prominence as a composite feed additive that combines prebiotic cell wall polysaccharides with postbiotic fermentation metabolites (Moonsamy et al., 2024). This dual nature enables a multi-target mode of action that is increasingly valued as an antibiotic alternative in managing infections caused by enteric pathogens. In the following sections, the present review dissect the barrier-centric mechanisms of yeast culture by component, highlighting how each contributes to intestinal defense and noting factors that influence efficacy.

### 4.1 Cell wall polysaccharides

Yeast cell wall polysaccharides–chiefly β-1,3/1,6-glucans and mannans/MOS–bolster the intestinal barrier through four complementary mechanisms. In terms of the mechanical barrier, dietary MOS improves epithelial TJs integrity. For example, adding 0.3% MOS in weanling pig diets upregulates TJs proteins, which reduces gut permeability and helps maintain villus architecture under ETEC challenge (Yu et al., 2021b). Strengthening TJs may limit the paracellular leak provoked by ETEC toxins, as intact junctions impede toxin entry and fluid loss. On the chemical barrier side, mannan-rich fractions stimulate mucus production. In trials, 500 mg/kg β-glucans supplementation increased the activities of maltase, sucrase, and mucosal enzymes in the ETEC-challenged pigs, and elevated *MUC2* expression in the intestine. A thicker mucus layer provides a larger buffer between pathogens and the epithelium, reducing bacterial adhesion and toxin contact (Zhou et al., 2022). By preserving mucus coverage, MOS may create a hostile environment for ETEC at the gut surface, effectively blunting toxin access to epithelial receptors. Related to the immune barrier, β-glucans and MOS interact with pattern-recognition receptors on immune cells to promote a balanced mucosal immune response. β-Glucans bind dectin-1 on intestinal dendritic cells, which skews responses toward Th2 immunity and increases sIgA production. Concurrently, MOS can engage Toll-like receptor 4 (TLR4) on macrophages, modulating nuclear factor- kappa B (NF-κB) signaling and inflammatory cytokine release. The net effect is sustained secretory sIgA output alongside a controlled cytokine milieu that protects the epithelium (Rehman et al., 2021; Vuscan et al., 2024). This immunomodulation yields a less inflammatory, more selective barrier that may fend off ETEC without collateral damage. At the microbial barrier layer, as prebiotics, MOS selectively feed beneficial microbes. Supplementation typically enriches *Lactobacillus* and *Bifidobacterium* populations in the gut and lowers luminal pH via their fermentation products (Jana et al., 2021). These changes may create an unfavorable setting for ETEC overgrowth. Importantly, MOS also functions as molecular decoys for ETEC fimbriae; their mannose-containing structures mimic host cell receptors, binding adhesins so that bacteria are blocked from attaching to the gut lining (Asadpoor et al., 2020). As the microbiota shifts toward health-promoting species, production of SCFAs like butyrate increases. Butyrate is a critical fuel for enterocytes and reinforces TJs while dampening local inflammation (Ghyselinck et al., 2025). This SCFA-mediated support may further fortify the epithelial barrier and assist in recovery from ETEC-induced injury.

Despite the overall positive trends, not all trials have found significant effects of yeast cell wall polysaccharides, largely due to variability in experimental conditions rather than a fundamental lack of efficacy. One issue is inconsistent product composition. Different β-glucans branching structures or MOS chain lengths can alter bioactivity, so two yeast products may not elicit the same immune response or adhesion-blocking effect (Zhao et al., 2023; Zheng et al., 2021b). Another factor is suboptimal application: efficacy is dose- and timing-dependent. Studies providing a sufficient MOS dose before and throughout an ETEC challenge (e.g., during the weaning period) tend to see clear benefits (Yu et al., 2021b), whereas under-dosing or starting supplementation only after infection often yields weak and no effects (Raymundo et al., 2024). The infection model and endpoints used also matter. Live ETEC challenges engage the full spectrum of adhesion and toxin effects that yeast components counter, whereas simpler lipopolysaccharide-only models can miss adhesion-blocking benefits (Browne et al., 2023; Yu et al., 2021b). Similarly, if a study only measures mRNA levels of TJs proteins and not actual barrier function, it may overlook improvements (yeast might strengthen junctions post-transcriptionally without changing mRNA) (Yu et al., 2021a). Baseline animal conditions play a role as well: young piglets with immature microbiota and immunity benefit more from MOS/β-glucans than older animals (Flores et al., 2024), and if the control diet already contains fermentable fiber or if pathogen pressure is low, the added benefit of yeast cell wall polysaccharides can be masked (Saliu et al., 2022). Even differences in ETEC strains or small sample sizes can lead to non-significant outcomes despite favorable trends (Dahmer et al., 2023; Fratto et al., 2024). In summary, these confounding factors (product differences, inadequate dosing, insensitive models or readouts, and varying initial conditions) explain most inconsistent findings. Recognizing them is important for interpreting the literature and does not undermine the general efficacy of yeast cell wall polysaccharides.

Despite some variability, several robust patterns emerge. Mannan-rich yeast products are particularly effective at blocking pathogen adhesion—ETEC-challenged piglets given such fractions show reduced bacterial attachment, lower gut permeability, and improved TJ integrity (Yu et al., 2021b). In contrast, β-glucans-enriched fractions excel at immunomodulation: they typically increase *MUC* expression and sIgA secretion while reducing pro-inflammatory cytokines, reflecting activation of dectin-1 and related pathways (Johnston et al., 2024). In practice, a MOS-focused yeast supplement might be chosen to target adhesion, whereas a β-glucans-rich supplement would be favored to broadly boost mucus production and immune protection. Importantly, when yeast-driven changes in the microbiota and its metabolites occur, barrier improvements often follow. Studies that reported an increase in butyrate-producing bacteria are usually the ones that also observed better TJ function, suggesting that fermentative metabolites from commensals help strengthen epithelial barriers and reduce inflammation (Li et al., 2025a). Therefore, measuring microbiota composition and SCFAs can be predictive; if a yeast supplement meaningfully increases butyrate-producers, one can expect barrier function gains to follow.

### 4.2 Metabolic products

Yeast postbiotic metabolic products fortify the intestine through a spectrum of barrier-focused actions. These soluble factors (organic acids, peptides, nucleotides, and polyphenols, etc.) operate at multiple layers. In terms of the mechanical barrier, postbiotics consistently promote a tighter epithelium. In nursery piglets, adding 175 g/t *Saccharomyces* postbiotics to the diet reduced early-weaning diarrhea and preserved villus architecture by stimulating crypt cell proliferation and activating mechanistic target of rapamycin signaling (Duarte and Kim, 2024). In vitro, a yeast metabolite blend protected intestinal cells from ETEC damage by maintaining higher transepithelial electrical resistance (TEER) during pathogen exposure (Tintoré et al., 2023). These findings imply that yeast-derived metabolites support rapid epithelial regeneration and prevent the dramatic TJ disruptions that ETEC would normally cause. In sow-fed and piglet-fed programs, 2.0 kg/mT fermentation-derived yeast postbiotics upregulated the piglets’ intestinal TJ genes and lowered the incidence of pre-weaning scours. Notably, these improvements in barrier function occurred without needing overt histological changes, suggesting that postbiotics improved junction assembly/function directly (Hung et al., 2025). Related to the immune barrier, yeast postbiotics help resolve inflammation and prevent collateral tissue damage during infection. In ETEC-challenged piglets, a 175 g/t *Saccharomyces* postbiotics diet led to lower *TLR4* expression in the gut lining and reduced levels of protein carbonylation in the mucosa. Treated piglets also had improved stool consistency (Gormley et al., 2024). These outcomes reflect a blunted danger response. In vitro immune assays likewise show that yeast metabolites shift macrophages toward an anti-inflammatory profile and enhanced phagocytic activity of immune cells (Carrera Marcolin et al., 2024), indicating a more effective but controlled immune response. Together, these data depict postbiotics as inflammation-modulators: they tamp down overzealous immune activation while supporting the clearance of pathogens. At the microbial layer, even though they contain no live microbes, yeast postbiotics can reshape the gut microbiota. During the ETEC challenge, piglets given yeast metabolites had higher abundances of beneficial genera like *Bifidobacterium* and *Corynebacterium*, along with less Gram-negative opportunists, and higher abundances of *Lactobacillus* populations, which produce lactic acid and other SCFAs that support gut health. These community shifts were linked to improved gut function and oxidative status in the host (Gormley et al., 2024). Essentially, postbiotics create a microbiome environment that is more resistant to pathogens and more nutritious for the host. The rise in SCFAs like butyrate contributes to the TJs and anti-inflammatory tone, complementing the direct effects of the postbiotics (Chen et al., 2025). In summary, these actions are mechanistically distinct from, but complementary to, yeast cell wall polysaccharides–together providing a multi-layered shield against ETEC.

Yeast postbiotics show a consistent barrier–protective profile in young animal models, often working through subtler molecular changes than cell wall components. Mechanistically, postbiotics differ from β-glucans/MOS by working through metabolic and signaling routes: for instance, limiting TLR4 signaling and activating cellular energy pathways (mechanistic target of rapamycin [mTOR]) for repair. These modes yield a phenotype of restrained innate activation and accelerated epithelial renewal (Gormley et al., 2024). In practical terms, young animals given yeast postbiotics tend to have better gut integrity during challenges–even if studies do not always detect a spike in barrier gene transcripts, improvements are reflected in better growth performance, lower oxidative damage, and reduced incidence of diarrhea. This highlights the importance of using appropriate endpoints: looking at intestinal permeability, redox markers, and clinical health gives a fuller picture of postbiotic benefits than transcriptional snapshots.

As with cell wall components, outcomes with yeast postbiotics can vary, and several key factors explain this heterogeneity. Firstly, postbiotics encompass a broad range of products: one formulation may contain a cocktail of organic acids, peptides, nucleotides, vitamins, etc. while another is more focused, so different studies may not be testing equivalent agents (Chae et al., 2024). This product-to-product variability means efficacy can differ simply because postbiotic A and postbiotic B differ in composition. Secondly, the challenge context influences what benefits are observed. In a highly inflammatory model, postbiotics clearly reduced TLR4 signaling and oxidative damage and boosted mTOR-mediated regeneration (Gormley et al., 2024), whereas in a milder weaning stress model without acute infection, the prominent effects were improved fecal scores and faster crypt cell proliferation with no change in TJ genes (Duarte and Kim, 2024). In both cases, the functional outcomes were less diarrhea and more resilience, but the measurable signals differed depending on whether the challenge was severe or mild. Thirdly, the sensitivity of the chosen assays can mask or reveal postbiotic effects. In vitro tests like TEER measurements or cell cytokine release assays often show significant improvements with yeast metabolites (Carrera Marcolin et al., 2024). In contrast, in vivo studies that only examine mucosal mRNA levels and have limited sample sizes might miss protein-level changes or functional improvements. For example, a non-significant trend in jejunal cytokine mRNA could mislead one to conclude no effect, even if the treated piglets clearly have less diarrhea and better growth (Duarte and Kim, 2024). Recognizing these nuances is important for interpreting why postbiotic studies sometimes differ. In summary, when the postbiotic product is well-defined, the challenge is appropriate, and the evaluation includes functional measures, studies consistently show that yeast postbiotics improve the intestinal barrier and animal health.

In aggregate, studies agree on several consistent outcomes of yeast postbiotic supplementation: (1) A nearly universal reduction in diarrhea incidence or severity is reported in ETEC-challenged animals given yeast metabolites (Gormley et al., 2024). (2) Treated animals often exhibit improved epithelial barrier function, such as higher TEER readings or lower pro-inflammatory secretions from gut tissues (Tintoré et al., 2023). (3) The mucosal environment becomes more anti-inflammatory–postbiotic-treated animals show reduced TLR4 activation and oxidative stress in the gut, and sometimes even lower systemic inflammatory cytokines, even if TJ gene up-regulation is not always observed (Gormley et al., 2024). Notably, fractionation studies indicate that the soluble metabolite fraction of yeast culture is the decisive factor behind these benefits. For example, adding a fermentation filtrate rich in yeast metabolites to pig diets improves TJ protein levels, whereas a comparable trial using only purified β-glucans did not (Zhen et al., 2021). Likewise, cell culture experiments showed that cell-free supernatants from yeast culture could enhance TEER and reduce inflammatory markers, mirroring the effects of the complete yeast product (Carrera Marcolin et al., 2024). This suggests that while β-glucans and MOS contribute to gut health, the postbiotic components are pivotal for maximal barrier reinforcement. Research points to a suite of small molecules as the active ingredients in yeast postbiotics, including SCFAs, organic acids, bioactive peptides, free nucleotides, vitamin-like cofactors, and antioxidant micronutrients produced during yeast fermentation (Chae et al., 2024). These diverse metabolites likely converge on key epithelial pathways–for instance, butyrate and other metabolites can activate energy and redox pathways in cells that accelerate repair and fortify tight junction assembly (Chen et al., 2025). Others may act as signaling molecules to dampen NF-κB inflammation or as direct substrates that improve the epithelial oxidative balance. The repeated observation that postbiotics up-regulate mTOR activity and down-regulate TLR4-driven signals supports this idea (Gormley et al., 2024). Future work isolating and testing specific metabolites would further clarify their individual roles, but current evidence underscores the importance of the whole metabolite cocktail for full efficacy. In summary, the metabolite-rich fraction of yeast culture is a major driver of barrier protection, working through a web of nutritional and signaling influences on the gut lining that complement the structural support from cell wall polysaccharides.

Taken together, yeast culture may strengthen intestinal defenses through complementary actions of cell-wall polysaccharides and postbiotic metabolites (Table 1). Although outcomes vary with product composition, dose, challenge model, and endpoints, consistent patterns show MOS excels at anti-adhesion, β-glucans at immunomodulation, and metabolite cocktails as key drivers of barrier protection. Standardizing characterization and incorporating functional endpoints will sharpen predictions of efficacy and support wider adoption in antibiotic-sparing programs across global herds and production systems.

**Table 1:** Experimental researches in young animals fed yeast culture components.

Supplement	Dosage	Experimental period	Animals	Effects	References
β-Glucan (28%)	0.05% diet	28 d	Weaned piglets (28 d of age)	Increased ADG; Increased VH/CD ratio in the jejunum and duodenum∗; Increased number of jejunal columnar epithelial cells∗; Decreased serum TNF-α and IL-1β concentrations∗; Upregulated mRNA expression of tight junction proteins (claudins and occludin) and *MUC1* in the jejunal mucosa∗; Increased serum IFN-γ and IL-6 concentrations∗; Increased relative abundance of *Prevotella* and *Roseburia* in the gut microbiota∗; Reduced diarrhea incidence.	Lee et al. (2021)
β-Glucan (60%)	500 mg/kg diet	28 d	Weaned piglets (21 d of age) ETEC challenge (experimental period d 25–28)	Increased villus height in the ileum, jejunum, and duodenum∗; Increased VH/CD ratio in the jejunum∗; Increased jejunal digestive enzyme activities (sucrase and maltase)∗; Increased relative abundance of *Lactobacillus* in intestinal contents∗; Increased colonic propionic acid concentration∗; Upregulated mRNA expression of jejunal *ZO-1* and *MUC2*∗	Zhou et al. (2022)
β-Glucan (90%)	65 mg/kg body weight	d 3 and 6	Neonatal calves (3 d of age)	Reduced diarrhea incidence∗; Decreased serum DAO activity∗; Increased fecal sIgA and defensin concentrations∗; Increased serum IL-6 concentration∗	Wang et al. (2025)
MOS (98.5%)	3 g/kg diet	21 d	Weaned piglets (21 d of age) ETEC challenge (experimental period d 19–21)	Reduced diarrhea incidence∗; Decreased fecal *E. coli* counts∗; Reduced serum D-lactic acid concentration and DAO activity∗; Increased jejunal ZO-1 protein expression∗; Increased intestinal sIgA concentration∗; Downregulated mRNA expression of jejunal *TNF-α*, *IL-1β*, and genes involved in the TLR4/NF-κB signaling pathway∗	Yu et al. (2021a)
MOS	14 mL/d	60 d	Neonatal calves (3 d of age)	Increased ADG∗; Reduced fecal *E. coli* counts∗; Decreased diarrhea incidence	Lucey et al. (2021)
MRF	0.1% diet	10 d	Weaned piglets (24 d of age) ETEC challenge (experimental period d 4, 5, and 10)	Increased ADG∗; Promoted intestinal lymphoid tissue hyperplasia∗; Reduced *E. coli* adhesion in the ileal mucosa;	Raymundo et al. (2024)
MRF	5 g/d	42 d	Neonatal calves (3 d of age)	Reduced diarrhea incidence∗; Increased villus height in the jejunum and colon∗; Increased acetic acid concentration in the jejunum and butyric acid concentration in the colon∗; Decreased serum IL-6 and TNF-α concentrations∗	Guo et al. (2025)
SYP	175 g/t diet	35 d	Weaned piglets (21 d of age)	Reduced diarrhea score∗; Increased jejunal crypt cell proliferation index∗; Increased cecal crypt depth; Upregulated mRNA expression of jejunal *mTOR* and *IFN-γ*	Duarte and Kim (2024)
SYP	175 g/t diet	28 d	Weaned piglets (21 d of age) ETEC challenge (experimental period d 7)	Reduced diarrhea score∗; Downregulated jejunal *TLR4* mRNA expression∗; Upregulated jejunal *mTOR* mRNA expression∗; Increased VH/CD ratio in the jejunum; Decreased relative abundance of gram-negative bacteria; Increased relative abundance of *Bifidobacterium*	Gormley et al. (2024)
Yeast peptide	1 g/d	d 21–35	Neonatal lambs (1 d of age)	Increased ADG; Increased VH/CD ratio in the jejunum∗; Increased relative abundance of *Lactobacillus* and *Aeriscardovia*∗	Li et al. (2023)
Yeast peptide	2000 mg/d	14 d	Neonatal lambs (1 d of age)	Reduced diarrhea incidence∗; Increased serum IL-4 and IL-10 concentrations∗; Decreased serum IL-1β and IL-6 concentrations∗; Upregulated mRNA expression of jejunal *MUC2*, claudin-1, claudin-4, occludin, and *ZO-1*∗; Increased relative abundance of *Roseburia*; Decreased relative abundance of *Staphylococcus* and *Escherichia*_*Shigella*	Fan et al. (2025)

MOS = mannan oligosaccharides; MRF = mannan-rich fraction; SYP = *Saccharomyces* yeast postbiotics; ETEC = enterotoxigenic *E*. *coli*; VH = villus height; CD = crypt depth; DAO = diamine oxidase; ADG = average daily gain; TNF-α = tumor necrosis factor-α; IL-1β = interleukin-1β; MUC1 = mucin 1; IFN-γ = interferon-γ; IL-6 = interleukin-6; MUC2 = mucin 2; ZO-1 = zonula occludens-1; sIgA = secretory immunoglobulin A; *E. coli* = *Escherichia coli*; TLR4 = Toll-like receptor 4; NF-κB = nuclear factor kappa-B; mTOR = mechanistic target of rapamycin; IL-4 = interleukin-4; IL-10 = interleukin-10. ∗, *P* < 0.05.

## 5 Practical value in neonatal livestock

Enterotoxigenic *E*. *coli*-associated diarrhea remains a leading cause of morbidity, growth setbacks, and mortality in neonatal and newly weaned livestock (Khalil et al., 2021). In an era of antibiotic reduction and heightened welfare standards, demonstrating that farms can control neonatal diarrhea with alternatives like yeast culture is strategically valuable. This review now translates the above mechanisms into practical outcomes observed in target species and provide practical insights for implementing yeast culture in neonatal livestock systems. Emphasis is given to piglets (as monogastric models) and calves/lambs (ruminant models), which together represent the major livestock affected by neonatal ETEC diarrhea.

### 5.1 Monogastric livestock

Piglets exemplify the monogastric livestock whose immature immune system and unstable gut microbiota render them highly susceptible to ETEC during birth and weaning (Tang et al., 2022). In both controlled ETEC challenges and on-farm trials, piglets fed yeast culture have exhibited significantly lower diarrhea rates and better growth than untreated piglets. Mechanistically, yeast β-glucans prime the piglet's immune defenses by activating innate cells and boosting sIgA (Han et al., 2020), while MOS and other yeast metabolites improve the piglet's antioxidant status and shield epithelial TJs from toxin-induced damage (Jiang et al., 2015; Yu et al., 2021a). Yeast supplementation also creates a healthier intestinal environment, improving gut morphology and shifting the microbiota toward beneficial species (more *Lactobacillus* and other butyrate-producers, fewer *E. coli*) (Zhou et al., 2022). These changes enhance nutrient absorption and colonization resistance. Notably, yeast-fed piglets frequently remain healthy even when ETEC bacteria are present at similar levels as in controls, indicating that yeast culture improves the host's disease tolerance rather than acting solely by reducing pathogen load (Cherrington et al., 2025). In essence, yeast culture helps piglets by strengthening host-centric protection – keeping their epithelial barrier intact and immune response balanced–so that ETEC cannot cause severe disease, even if some bacteria are still present. This host-focused fortification is what yields more robust, fast-growing piglets in the face of ETEC risk.

Across controlled experiments and farm trials, yeast culture consistently lowers post-weaning diarrhea in piglets by 40% to 60%, with benefits most evident during the highest-risk weeks. In a 28-d nursery study, yeast cell-wall supplementation virtually eliminated early-week scours and reduced week-2 cases versus controls, while delivering 6% to 7% higher average daily gain (Lee et al., 2021). In ETEC challenge models, yeast culture achieved diarrhea control comparable to medicated groups using antibiotics or high-dose ZnO, and it helped maintain normal gut permeability and TJ markers (Che et al., 2017). Performance gains are context-dependent: under high pathogen load or sanitary stress, yeast limits diarrhea-related growth checks and modestly lifts average daily gain; in low-challenge, ultra-clean conditions, fecal scores still improve, but growth advantages often narrow and may not reach significance (Smallfield et al., 2025). Overall, the evidence supports yeast culture as a pragmatic health insurance that consistently improves digestive health and frequently sustains growth when stressors would otherwise depress performance.

To apply these findings in practice, producers should focus on the following suggestions: better results are obtained by introducing yeast culture before and during the weaning period, feeding it continuously at an effective dose, and selecting a product appropriate to the farm's challenge (for instance, MOS-rich products are preferred if ETEC adhesion is the main concern, or metabolite-rich products if post-weaning inflammation is the greater issue). Economically, yeast culture yields the highest return where diarrhea risk is moderate to high. Under ETEC pressure, its low inclusion cost is outweighed by savings from fewer growth setbacks and reduced antibiotic use (Kim et al., 2022). Ficagna et al. (2025) showed that MOS-based additives improved feed efficiency, minimized fallback pigs, and cut waste during health slumps, resulting in clear profit margins. Healthy guts also enhance nutrient absorption, compounding performance gains. In well-managed, low-challenge farms, returns are narrower, yet many producers still apply yeast culture as preventive insurance for herd uniformity and resilience (Zheng et al., 2021a). Because yeast is included at small dietary rates, it rarely affects feed intake or taste. Trials report no palatability issues, and piglets consume it readily in creep or starter feeds, maintaining appetite and steady growth through the stress-prone weaning transition (Huenul et al., 2023). In practical terms, yeast culture is user-friendly and well-suited to swine diets: it comes as a dry, shelf-stable ingredient that can be easily mixed into standard rations and pelleted feed. These advantages underscore the economic appeal of yeast culture. By improving feed efficiency and survival rates, yeast culture ultimately helps producers recoup its cost through healthier, faster-growing piglets and fewer disease-related setbacks.

Finally, it's important to place yeast culture in the context of overall herd management. Yeast culture is a valuable tool to enhance gut health and reduce diarrhea, but it is not a magic bullet that can overcome fundamentally poor management. Yeast culture works best when basic husbandry is sound, and it cannot compensate for failures in colostrum provision, hygiene, or nutrition. Indeed, the largest yeast culture gains have been observed on farms that also excel in these fundamental areas of neonatal care (Vangroenweghe and Boone, 2022). Under such conditions, yeast culture proves to be a practical, regulation-friendly strategy to reduce ETEC diarrhea in piglets, aligning with efforts to curb antibiotic and zinc use. It is easy to implement via feed and has demonstrated efficacy in research and field settings, making it a clear success case and paving the way for similar barrier-focused interventions in calves and other youngstock.

### 5.2 Ruminant livestock

Like piglets, neonatal calves and lambs are highly susceptible to ETEC during their first weeks of life – especially before the rumen fully develops and while their immune system is immature. Yeast culture provides these young ruminants with a broad-spectrum, antibiotic-free safeguard in this vulnerable window. This aligns perfectly with the dairy/beef/mutton sector's shift away from routine antibiotic use in ruminant livestock-rearing. Consistent evidence shows that feeding yeast culture from birth through weaning improves calf and lamb intestinal barrier integrity, boosts their immune readiness, and steadies their gut microbiota. For instance, calves on yeast culture have been observed to develop healthier intestinal morphology with stronger TJ protein expression than controls (Li et al., 2023). They also exhibit a more responsive yet balanced mucosal immune profile – notably higher sIgA levels and well-regulated innate responses (Wang et al., 2025). Additionally, yeast postbiotic-fed ruminant neonates develop gut microbial communities that are less hospitable to pathogens, often showing more beneficial fermenters and fewer opportunistic *E. coli* blooms (Centeno-Martinez et al., 2023). Together, these changes translate into tangible benefits: better growth rates, less dehydration, and higher survival when calves or lambs face ETEC or similar enteric threats.

A Meta-analysis of 34 studies in China put average pre-weaning diarrhea prevalence in calves at around 27% (Qu et al., 2023), highlighting how common and costly scours are – exactly the issue yeast culture is meant to mitigate. One controlled trial in dairy calves found that supplementing a mannan-rich yeast fraction reduced pre-weaning diarrhea incidence by 36% and increased body weight gain by 6% compared with unsupplemented calves. Treated calves also showed higher levels of beneficial SCFAs and taller villi in the intestine, along with lower inflammatory cytokines, indicating improved gut health and recovery (Guo et al., 2025). The MOS in yeast culture likely acted as a decoy for ETEC fimbriae – binding the bacteria and preventing them from attaching to the calf's gut, thereby reducing pathogen colonization and toxin delivery (Asbury and Saville, 2025). Likewise, in a large dairy herd trial, calves given yeast culture had higher pre-weaning weight gain and shed fewer intestinal pathogens, although the duration of scours was not significantly changed (Lucey et al., 2021). Overall, controlled studies and farm trials consistently show that calves receiving yeast culture experience lower scour rates and better growth than those without.

There is less research in lambs than in calves, but emerging studies show comparable benefits and mechanisms. In a controlled study, lambs given yeast-derived peptides had dose-dependent reductions in diarrhea and better fecal consistency, with the highest dose (2000 mg/d) producing the greatest benefit. Treated lambs also exhibited higher expression of TJ proteins (occluding and claudin-4) and MUC2, indicating a stronger intestinal barrier, and their microbiota shifted toward more beneficial fermenters (e.g., increased *Roseburia*) and fewer pathogens. Over a 14-d trial, weight gain was similar between groups (not unexpected given the short duration), but importantly, the yeast peptide groups experienced significantly fewer scours in that critical neonatal period (Fan et al., 2025). These findings highlight the potential of targeted yeast fractions (like peptides) in ruminant neonates.

Outside the high-risk neonatal phase, yeast culture still offers notable benefits for growing lambs and sheep. Various studies in weaned lambs and feedlot sheep have found that adding yeast culture to the diet can improve feed intake and growth rates, promote better rumen development, and increase feed digestibility (Li et al., 2024; Xu et al., 2025). These enhancements suggest that yeast culture creates a more stable and efficient gut fermentation ecosystem in young ruminants. This stability can indirectly enhance the animal's resilience to enteric pathogens. While direct ETEC challenge trials in older lambs are still lacking, it's plausible that the same yeast-driven improvements–stronger nutrient absorption, consistent rumen pH, a robust population of beneficial microbes–help keep pathogens in check. Thus, beyond preventing neonatal scours, yeast culture contributes to a robust gut overall, which is the foundation of health and performance.

Outcomes with yeast culture in young ruminants can differ due to a range of factors. The impact of yeast cultures is more pronounced when the neonate's immunity is weak or when rearing conditions are highly stressful. For example, lambs in many systems are weaned very early and often face cold outdoor conditions or are reared as twins/triplets, which can stress them and elevate disease risk (Shi et al., 2022), whereas dairy calves raised in groups on milk replacer encounter different challenges such as sanitation of feeding equipment, variability in milk mixing, and risks of cross-suckling (Welk et al., 2023). These differing early-life stress and exposure scenarios influence how much room there is for an additive to help, with yeast culture yielding big benefits in high-stress environments by fortifying their gut defenses but offering only subtle improvements on well-immunized, low-stress farms (Wang et al., 2023). Variation in the yeast product and its use also plays a role: different yeast strains or preparations, as well as dosage and timing, can lead to inconsistent results if not optimized. In general, calves and lambs both respond positively to yeast supplementation, but understanding these species differences, immune and management factors, and product considerations allows one to tailor its use for maximal effect in each situation.

The key to success with yeast culture in calves and lambs mirrors that highlighted for piglets: it should be started early, used consistently, and integrated into good management practices. In practical terms, producers should likewise introduce yeast culture from birth and continue it daily until weaning, in conjunction with good neonatal care (Li et al., 2025b). Yeast culture works best as a supplement to sound husbandry, not a substitute for it (Constable et al., 2021). Farms should monitor scour incidence and track growth rates to assess success (Edwards and Renaud, 2025), and use diagnostics to confirm whether an outbreak is ETEC or another pathogen (Sedky et al., 2025). When used in conjunction with proper colostrum intake, sanitation, and housing, yeast culture may decrease scour incidence and severity and improve early growth and vigor in young ruminants. Notably, yeast culture enables producers to curb antibiotic use by preventing disease in the first place, aligning with antimicrobial stewardship goals and improving animal welfare. In summary, yeast culture offers a multifaceted, policy-aligned solution to neonatal diarrhea, allowing calves and lambs to be raised with greater resilience and minimal antibiotic intervention.

## 6 Future perspectives

Despite promising progress, several critical knowledge gaps remain. (1) Further research is required to clarify which components of yeast culture provide the greatest protective effects, including the precise receptor-mediated pathways associated with different β-glucans structures or mannan fractions. Advanced tools (e.g., gut organoids, transcriptomics) should be applied to elucidate how these components interact with the host at the molecular level. (2) Optimal dosing and formulation: The field lacks consensus on ideal dosing regimens and formulations for various species. The minimum effective dose in piglets compared with calves remains to be clearly defined, and the influence of yeast strain and processing method on efficacy requires systematic evaluation. Controlled comparative trials are needed to establish standardized dosing strategies and to assess shelf-stable formulations, enabling reliable on-farm implementation. (3) Species-specific protocols: neonatal pigs, calves, and lambs differ in digestive physiology and maternal immunity; consequently, yeast culture protocols may require species-specific adaptation. Research should determine how yeast culture use can be customized—such as incorporation into milk replacers for calves versus creep feed for piglets—to maximize benefits during critical early-life periods. (4) Integrated disease management: finally, the potential for yeast culture to function synergistically with other interventions warrants systematic investigation. The combined use of yeast culture with targeted probiotics, vaccines, or nutritional adjustments should be evaluated for additive or complementary protective effects. Exploration of such integrative approaches may facilitate the development of comprehensive gut health programs that reduce reliance on antibiotics.

Embracing yeast culture is more than a single-solution strategy–it is a step toward a holistic, preventative healthcare model in animal production. By bolstering the animal's own defenses, reliance on antibiotics and pharmaceuticals can be reduced, thereby addressing the twin challenges of antimicrobial resistance and drug residues in the food chain. In the coming years, yeast culture is anticipated to serve as a cornerstone of antibiotic-free rearing systems, supported by rigorous research-driven protocols. This will involve not only refining the biological understanding as outlined above, but also demonstrating economic viability and consistency in the field. Achieving these goals will directly contribute to sustainable livestock farming–healthier animals, improved welfare, and enhanced food safety for consumers. Ultimately, reinforcing the intestinal barrier through innovations like yeast culture provides a proactive path to disease prevention, aligning livestock production with the global mandate for sustainability and antibiotic stewardship.

## 7 Conclusion

This review highlights that strengthening the host's own intestinal barrier is a viable and effective strategy for mitigating ETEC-induced diarrhea in neonatal livestock. By focusing on barrier-targeted mechanisms, the perspective shifts from the traditional pathogen-centric approach to one of enhancing host resilience. Yeast culture, with its combined prebiotic and postbiotic features, exemplifies this paradigm: rather than killing bacteria directly, it empowers the host to withstand infection by fortifying TJs, enhancing mucus production, modulating immunity, and supporting a healthy microbiome. Embracing such a barrier-centric approach could markedly reduce antibiotic dependence in livestock production, aligning with broader efforts toward sustainable, antibiotic-free animal agriculture. With continued research to refine usage and understand its multifaceted actions, yeast culture can be integrated as a key component of holistic herd health management. Ultimately, bolstering the intestinal barrier through interventions like yeast culture offers a proactive path to healthier livestock and a more sustainable agriculture–one where disease prevention is achieved by reinforcing the very defenses that nature built into the host.

## Credit Author Statement

**Shuai Wang:** Writing – original draft, Visualization, Investigation. **Pengxiang Bai:** Investigation, Conceptualization. **Hui Chen:** Investigation, Conceptualization. **Lan Yang:** Writing – review & editing, Conceptualization. **Dacheng Liu:** Supervision, Project administration, Conceptualization.

## Declaration of competing interest

We declare that we have no financial and personal relationships with other people or organizations that can inappropriately influence our work, and there is no professional or other personal interest of any nature or kind in any product, service and/or company that could be construed as influencing the content of this paper.

## References

[bib1] Asadpoor M., Peeters C., Henricks P.A., Varasteh S., Pieters R.J., Folkerts G., Braber S. (2020). Anti-pathogenic functions of non-digestible oligosaccharides in vitro. Nutrients.

[bib2] Asbury R.E., Saville B.A. (2025). Manno-oligosaccharides as a promising antimicrobial strategy: pathogen inhibition and synergistic effects with antibiotics. Front Microbiol.

[bib3] Barros M.M., Campos A.M., Castro J., Oliveira R., Araújo D., Outor-Monteiro D., Almeida C. (2025). An asset for food safety: the knowledge behind the physiological alterations induced by etec enterotoxins. Foods.

[bib4] Bin P., Tang Z., Liu S., Chen S., Xia Y., Liu J., Wu H., Zhu G. (2018). Intestinal microbiota mediates enterotoxigenic *Escherichia coli*-induced diarrhea in piglets. BMC Vet Res.

[bib5] Browne N., Daly D., Horgan K. (2023). Differential impact of yeast cell wall products in recovery of porcine intestinal epithelial cell barrier function following lipopolysaccharide challenge. Porcine Health Manag.

[bib6] Cai R., Cheng C., Chen J., Xu X., Ding C., Gu B. (2020). Interactions of commensal and pathogenic microorganisms with the mucus layer in the colon. Gut Microbes.

[bib7] Carrera Marcolin L., Cuñé Castellana J., Martí Melero L., De Lecea C., Tintoré Gazulla M. (2024). Synergistic effect of postbiotic yeast abb c22® on gut inflammation, barrier function, and protection from rotavirus infection in in vitro models. Appl Microbiol.

[bib8] Centeno-Martinez R.E., Dong W., Klopp R.N., Yoon I., Boerman J.P., Johnson T.A. (2023). Effects of feeding *saccharomyces cerevisiae* fermentation postbiotic on the fecal microbial community of holstein dairy calves. Animal Microbiome.

[bib9] Chae J.-B., Schoofs A.D., Mcgill J.L. (2024). Beneficial effects of *saccharomyces cerevisiae* fermentation postbiotic products on calf and cow health and plausible mechanisms of action. Front Anim Sci.

[bib10] Che L., Xu Q., Wu C., Luo Y., Huang X., Zhang B., Auclair E., Kiros T., Fang Z., Lin Y. (2017). Effects of dietary live yeast supplementation on growth performance, diarrhoea severity, intestinal permeability and immunological parameters of weaned piglets challenged with enterotoxigenic *Escherichia coli* k88. Br J Nutr.

[bib11] Chen W., Ma Q., Li Y., Wei L., Zhang Z., Khan A., Khan M.Z., Wang C. (2025). Butyrate supplementation improves intestinal health and growth performance in livestock: a review. Biomolecules.

[bib12] Cherrington T., Jordan D., Pluske J., Mansfield J., Lugsomya K., Wilkinson S., Cadogan D., Abraham S., O’dea M. (2025). *Lactobacillus* and *saccharomyces* fermentation products impact performance and the fecal microbiome in weanling pigs inoculated with enterotoxigenic *Escherichia coli*. J Anim Sci.

[bib13] Constable P.D., Trefz F.M., Sen I., Berchtold J., Nouri M., Smith G., Grünberg W. (2021). Intravenous and oral fluid therapy in neonatal calves with diarrhea or sepsis and in adult cattle. Front Vet Sci.

[bib14] Dahmer P.L., Derouchey J.M., Gebhardt J.T., Paulk C.B., Jones C.K. (2023). Summary of methodology used in enterotoxigenic *Escherichia coli* (etec) challenge experiments in weanling pigs and quantitative assessment of observed variability. Transl Anim Sci.

[bib15] Duarte M.E., Kim S.W. (2024). Efficacy of *saccharomyces* yeast postbiotics on cell turnover, immune responses, and oxidative stress in the jejunal mucosa of young pigs. Sci Rep.

[bib16] Dubreuil J.D. (2017). Enterotoxigenic *Escherichia coli* targeting intestinal epithelial tight junctions: an effective way to alter the barrier integrity. Microb Pathog.

[bib17] Dubreuil J.D., Isaacson R.E., Schifferli D.M., Donnenberg M.S. (2016). Animal enterotoxigenic *Escherichia coli*. EcoSal Plus.

[bib18] Duysburgh C., Miclotte L., Green J.B., Watts K.T., Sardi M.I., Chakrabarti A., Khafipour E., Marzorati M. (2024). *Saccharomyces cerevisiae* derived postbiotic alters gut microbiome metabolism in the human distal colon resulting in immunomodulatory potential in vitro. Front Microbiol.

[bib19] Edwards K.Y., Renaud D.L. (2025). A framework for comprehensive dairy calf health investigations. Animals.

[bib20] Fan D., Zong R., Zhang C., Zhang J., Chai J., Cui K., Zhang N. (2025). Yeast peptides alleviate diarrhea in neonatal lambs by enhancing the colonic barrier function and modulating colonic microbiota. Front Vet Sci.

[bib21] Faustino M., Durão J., Pereira C.F., Pintado M.E., Carvalho A.P. (2021). Mannans and mannan oligosaccharides (mos) from *saccharomyces cerevisiae*–a sustainable source of functional ingredients. Carbohydr Polym.

[bib22] Ficagna C.A., Silva A.S.D., Rofino R.D., Zatti E., Esposito T., Xavier A.C.H., Wagner R., Bissacotti B.F., Seghetto R.B., Ternus E.M. (2025). Effects on performance, immunological response and short-chain fatty acid profile in feces of nursery piglets fed with organic acids and yeast wall. Animals.

[bib23] Flores J.N., Lubin J.-B., Silverman M.A. (2024). The case for microbial intervention at weaning. Gut Microbes.

[bib24] Fratto A., Torricelli M., Sebastiani C., Ciullo M., Felici A., Biagetti M. (2024). Survey on resistance occurrence for f4+ and f18+ enterotoxigenic *Escherichia coli* (etec) among pigs reared in central Italy regions. Vet Res Commun.

[bib25] Ghyselinck J., Verstrepen L., Rakebrandt M., Marynissen S., Daminet S., Marzorati M. (2025). In vitro fermentation of yeast cell walls (mannan-oligosaccharide) and purified β-glucans modulates the colonic microbiota of dogs with inflammatory bowel disease and demonstrates protective effects on barrier integrity and anti-inflammatory properties. PLoS One.

[bib26] Gieryńska M., Szulc-Dąbrowska L., Struzik J., Mielcarska M.B., Gregorczyk-Zboroch K.P. (2022). Integrity of the intestinal barrier: the involvement of epithelial cells and microbiota—a mutual relationship. Animals.

[bib27] Gormley A.R., Duarte M.E., Deng Z., Kim S.W. (2024). *Saccharomyces* yeast postbiotics mitigate mucosal damages from f18+ *Escherichia coli* challenges by positively balancing the mucosal microbiota in the jejunum of young pigs. Anim Microb.

[bib28] Gryaznova M.V., Dvoretskaya Y.D., Syromyatnikov M.Y., Shabunin S.V., Parshin P.A., Mikhaylov E.V., Strelnikov N.A., Popov V.N. (2022). Changes in the microbiome profile in different parts of the intestine in piglets with diarrhea. Animals.

[bib29] Guo S., Feng Y., Yang J., Zhao H., Ma J., Zhang Y., Sun M., Li Y., Lin G., Lin P., Wang A., Jin Y. (2025). Mannan-rich fraction supplementation: a promising nutritional strategy for optimizing growth and health of pre-weaning calves. Animals.

[bib30] Han B., Baruah K., Cox E., Vanrompay D., Bossier P. (2020). Structure-functional activity relationship of β-glucans from the perspective of immunomodulation: a mini-review. Front Immunol.

[bib31] Higashi T., Saito A.C., Chiba H. (2024). Damage control of epithelial barrier function in dynamic environments. Eur J Cell Biol.

[bib32] Higginson E.E., Sayeed M.A., Pereira Dias J., Shetty V., Ballal M., Srivastava S.K., Willis I., Qadri F., Dougan G., Mutreja A. (2022). Microbiome profiling of enterotoxigenic *Escherichia coli* (etec) carriers highlights signature differences between symptomatic and asymptomatic individuals. mBio.

[bib33] Huenul E., Salazar L., Frias D., Videka M., Luna D., Dwyer D.M., Figueroa J. (2023). Effects of flavour variety on the intake and palatability of commercial feed in nursery pigs. Front Vet Sci.

[bib34] Hung P.H.S., Thi Dung H., Thao L.D., Van Chao N., Thi Hoa N., Thi Hien B., Mondal A., Nsereko V., Phung L.D. (2025). Effects of *saccharomyces cerevisiae* fermentation-derived postbiotics supplementation in sows and piglets' diet on intestinal morphology, and intestinal barrier function in weaned pigs in an intensive pig production system. Vet Immunol Immunopathol.

[bib35] Ikewaki N., Iwasaki M., Kurosawa G., Rao K.-S., Lakey-Beitia J., Preethy S., Abraham S.J. (2021). Β-glucans: wide-spectrum immune-balancing food-supplement-based enteric (β-wife) vaccine adjuvant approach to covid-19. Hum Vacc Immunother.

[bib36] Jana U.K., Kango N., Pletschke B. (2021). Hemicellulose-derived oligosaccharides: emerging prebiotics in disease alleviation. Front Nutr.

[bib37] Jiang Z., Wei S., Wang Z., Zhu C., Hu S., Zheng C., Chen Z., Hu Y., Wang L., Ma X. (2015). Effects of different forms of yeast *saccharomyces cerevisiae* on growth performance, intestinal development, and systemic immunity in early-weaned piglets. J Anim Sci Biotechnol.

[bib38] Johnston C.J.H., Ledwith A.E., Lundahl M.L., Charles-Messance H., Hackett E.E., O’shaughnessy S.D., Clegg J., Prendeville H., Mcgrath J.P., Walsh A.M. (2024). Recognition of yeast β-glucan particles triggers immunometabolic signaling required for trained immunity. iScience.

[bib39] Khalil I., Walker R., Porter C.K., Muhib F., Chilengi R., Cravioto A., Guerrant R., Svennerholm A.-M., Qadri F., Baqar S. (2021). Enterotoxigenic *escherichia coli* (etec) vaccines: priority activities to enable product development, licensure, and global access. Vaccine.

[bib40] Kim K., Song M., Liu Y., Ji P. (2022). Enterotoxigenic *escherichia coli* infection of weaned pigs: intestinal challenges and nutritional intervention to enhance disease resistance. Front Immunol.

[bib41] Laird T.J., Abraham S., Jordan D., Pluske J.R., Hampson D.J., Trott D.J., O’dea M. (2021). Porcine enterotoxigenic *escherichia coli*: antimicrobial resistance and development of microbial-based alternative control strategies. Vet Microbiol.

[bib42] Leclercq E., Pontefract N., Rawling M., Valdenegro V., Aasum E., Andujar L.V., Migaud H., Castex M., Merrifield D. (2020). Dietary supplementation with a specific mannan-rich yeast parietal fraction enhances the gut and skin mucosal barriers of atlantic salmon (salmo salar) and reduces its susceptibility to sea lice (lepeophtheirus salmonis). Aquaculture.

[bib43] Lee J.J., Kyoung H., Cho J.H., Choe J., Kim Y., Liu Y., Kang J., Lee H., Kim H.B., Song M. (2021). Dietary yeast cell wall improves growth performance and prevents of diarrhea of weaned pigs by enhancing gut health and anti-inflammatory immune responses. Animals.

[bib44] Li J., Liu X., Sun C., Wang M., Zhang Q., Wang H., Ji X., Jin E., Zhang F. (2025). Metabolomics reveals the hindgut metabolic changes and physiological impacts in weaned piglets subjected to etec infection and berberine intervention. Sci Rep.

[bib45] Li X., Wang Y., Xu J., Yang Q., Sha Y., Jiao T., Zhao S. (2024). Effects of yeast cultures on meat quality, flavor composition and rumen microbiota in lambs. Curr Res Food Sci.

[bib46] Li X., Yang X., Liu S., Liang X., Chen H., Liu D. (2025). Yeast culture improves growth, antioxidant status, immunity, and gut microbiota homeostasis in preweaning holstein calves. Front Vet Sci.

[bib47] Li Y., Han L., Liu J., Kang L., Zhao L., Cui K. (2023). Yeast peptides improve the intestinal barrier function and alleviate weaning stress by changing the intestinal microflora structure of weaned lambs. Microorganisms.

[bib48] Liu B., Wang W., Deng Z., Ma C., Wang N., Fu C., Lambert H., Yan F. (2023). Antibiotic governance and use on commercial and smallholder farms in eastern China. Front Vet Sci.

[bib49] Liu S., Yang L., Zhang Y., Chen H., Li X., Xu Z., Du R., Li X., Ma J., Liu D. (2024). Review of yeast culture concerning the interactions between gut microbiota and young ruminant animals. Front Vet Sci.

[bib50] Lucey P., Lean I., Aly S., Golder H., Block E., Thompson J., Rossow H. (2021). Effects of mannan-oligosaccharide and bacillus subtilis supplementation to preweaning holstein dairy heifers on body weight gain, diarrhea, and shedding of fecal pathogens. J Dairy Sci.

[bib51] Luo Q., Kumar P., Vickers T.J., Sheikh A., Lewis W.G., Rasko D.A., Sistrunk J., Fleckenstein J.M. (2014). Enterotoxigenic *escherichia coli* secretes a highly conserved mucin-degrading metalloprotease to effectively engage intestinal epithelial cells. Infect Immun.

[bib52] Luo Q., Yang L., Tumenjargal B., Liu S., Ma J., Ning J., Yun Z., Zhang X., Wu Y., Lu Y. (2024). Effect of composite yeast culture on the jejunal barrier function, inflammatory response, and microbial community structure of laying hens during the late stage of egg production. Front Vet Sci.

[bib53] Maturana M., Castillejos L., Martin-Orue S.M., Minel A., Chetty O., Felix A.P., Adib Lesaux A. (2023). Potential benefits of yeast *saccharomyces* and their derivatives in dogs and cats: a review. Front Vet Sci.

[bib54] Menge C. (2020). The role of *escherichia coli* shiga toxins in stec colonization of cattle. Toxins.

[bib55] Moonsamy G., Roets-Dlamini Y., Langa C.N., Ramchuran S.O. (2024). Advances in yeast probiotic production and formulation for preventative health. Microorganisms.

[bib56] Mooyottu S., Muyyarikkandy M.S., Yousefi F., Li G., Sahin O., Burrough E., Scaria J., Sponseller B., Ramirez A. (2025). Fecal microbiota transplantation modulates jejunal host-microbiota interface in weanling piglets. Microbiome.

[bib57] Nam J., Kim A., Kim K., Moon J.H., Baig J., Phoo M., Moon J.J., Son S. (2024). Engineered polysaccharides for controlling innate and adaptive immune responses. Nat Rev Bioeng.

[bib58] Nielsen J.O., Aarestrup F.M., Andersen V.D., Vigre H. (2025). The effect of the discontinued use of zinc oxide on antimicrobial usage in danish pig farms. Prev Vet Med.

[bib59] Niewiem M., Grzybowska-Chlebowczyk U. (2022). Intestinal barrier permeability in allergic diseases. Nutrients.

[bib60] Osorio J.S. (2020). Gut health, stress, and immunity in neonatal dairy calves: the host side of host-pathogen interactions. J Anim Sci Biotechnol.

[bib61] Pang Y., Zhang H., Wen H., Wan H., Wu H., Chen Y., Li S., Zhang L., Sun X., Li B., Liu X. (2022). Yeast probiotic and yeast products in enhancing livestock feeds utilization and performance: an overview. J Fungi.

[bib62] Park J.-Y., Abekura F., Cho S.-H. (2023). Gm1a ganglioside-binding domain peptide inhibits host adhesion and inflammatory response of enterotoxigenic *escherichia coli* heat-labile enterotoxin-b in hct-8 cells. Sci Rep.

[bib63] Patra A.K., Amasheh S., Aschenbach J.R. (2019). Modulation of gastrointestinal barrier and nutrient transport function in farm animals by natural plant bioactive compounds–a comprehensive review. Crit Rev Food Sci Nutr.

[bib64] Patterson R., Rogiewicz A., Kiarie E.G., Slominski B.A. (2023). Yeast derivatives as a source of bioactive components in animal nutrition: a brief review. Front Vet Sci.

[bib65] Qu J., Meng C., Li L., Zhu H., Yin G. (2023). Epidemiology of diarrhea for Chinese dairy calves: a systematic review and meta-analysis. Indian J Anim Res.

[bib66] Raymundo D.L., Borges P.C., Barbosa K., Utiumi K.U., Varaschin M.S., Leal D.F., Silva Jr SR., Resende M., Barbosa J.A., De Souza Cantarelli V. (2024). Effects of dietary yeast mannan-rich fraction supplementation on growth performance, intestinal morphology, and lymphoid tissue characteristics in weaned piglets challenged with *escherichia coli* f4. Trop Anim Health Prod.

[bib67] Rehman M.S.-U., Rehman S.U., Yousaf W., Hassan F.-U., Ahmad W., Liu Q., Pan H. (2021). The potential of toll-like receptors to modulate avian immune system: exploring the effects of genetic variants and phytonutrients. Front Genet.

[bib68] Rogers A.P., Mileto S.J., Lyras D. (2023). Impact of enteric bacterial infections at and beyond the epithelial barrier. Nat Rev Microbiol.

[bib69] Rooks M.G., Garrett W.S. (2016). Gut microbiota, metabolites and host immunity. Nat Rev Immunol.

[bib70] Saliu E.-M., Martínez-Vallespín B., Aschenbach J., Brockmann G., Fulde M., Hartmann S., Kuhla B., Lucius R., Metges C., Rothkötter H. (2022). Dietary fiber and its role in performance, welfare, and health of pigs. Anim Health Res Rev.

[bib71] Sauvaitre T., Van Landuyt J., Durif C., Roussel C., Sivignon A., Chalancon S., Uriot O., Van Herreweghen F., Van De Wiele T., Etienne-Mesmin L. (2022). Role of mucus-bacteria interactions in enterotoxigenic *Escherichia coli* (etec) h10407 virulence and interplay with human microbiome. npj Biofilms Microb.

[bib72] Sedky D., Ghazy A.A., Abou-Zeina H.A. (2025). Advances in diagnosis of diseases causing diarrhea in newborn calves. Vet Res Commun.

[bib73] Serek P., Oleksy-Wawrzyniak M. (2021). The effect of bacterial infections, probiotics and zonulin on intestinal barrier integrity. Int J Mol Sci.

[bib74] Sheikh A., Wangdi T., Vickers T.J., Aaron B., Palmer M., Miller M.J., Kim S., Herring C., Simoes R., Crainic J.A. (2022). Enterotoxigenic *Escherichia coli* degrades the host muc2 mucin barrier to facilitate critical pathogen-enterocyte interactions in human small intestine. Infect Immun.

[bib75] Shi L., Xu Y., Jin X., Wang Z., Mao C., Guo S., Yan S., Shi B. (2022). Influence of cold environments on growth, antioxidant status, immunity and expression of related genes in lambs. Animals.

[bib76] Smallfield J.L., Derouchey J.M., Tokach M.D., Woodworth J.C., Goodband R.D., Gaffield K.N., Gebhardt J.T., Yao R., Guo Y. (2025). Effects of hydrolyzed yeast on weanling pig growth performance, fecal dry matter, and stress-related blood antioxidant criteria. J Anim Sci.

[bib77] Su W., Gong T., Jiang Z., Lu Z., Wang Y. (2022). The role of probiotics in alleviating postweaning diarrhea in piglets from the perspective of intestinal barriers. Front Cell Infect Microbiol.

[bib78] Tang X., Xiong K., Fang R., Li M. (2022). Weaning stress and intestinal health of piglets: a review. Front Immunol.

[bib79] Tao E., Lang D. (2024). Unraveling the gut: the pivotal role of intestinal mechanisms in Kawasaki disease pathogenesis. Front Immunol.

[bib80] Tintoré M., Cuñé J., Vetvicka V., De Lecea C. (2023). Anti-inflammatory effects, protection of gut barrier integrity and stimulation of phagocytosis of postbiotic combination abb c1. Nutraceuticals.

[bib81] Vangroenweghe F.A., Boone M. (2022). Vaccination with an *Escherichia coli* f4/f18 vaccine improves piglet performance combined with a reduction in antimicrobial use and secondary infections due to Streptococcus suis. Animals.

[bib82] Von Mentzer A., Svennerholm A.-M. (2024). Colonization factors of human and animal-specific enterotoxigenic *Escherichia coli* (etec). Trends Microbiol.

[bib83] Vuscan P., Kischkel B., Hatzioannou A., Markaki E., Sarlea A., Tintoré M., Cuñé J., Verginis P., De Lecea C., Chavakis T. (2024). Potent induction of trained immunity by *Saccharomyces cerevisiae* β-glucans. Front Immunol.

[bib84] Wang J., Ji H. (2019). Tight junction proteins in the weaned piglet intestine: roles and regulation. Curr Protein Pept Sci.

[bib85] Wang J., Yan F., Xiong M., Dong J., Yang W., Xu X. (2025). Effects of yeast β-glucan supplementation on calf intestinal and respiratory health. Animals.

[bib86] Wang L., Sun H., Gao H., Xia Y., Zan L., Zhao C. (2023). A meta-analysis on the effects of probiotics on the performance of pre-weaning dairy calves. J Anim Sci Biotechnol.

[bib87] Wang S., Zhu S., Zhang J., Li H., Yang D., Huang S., Wei Z., Liang X., Wang Z. (2020). Supplementation with yeast culture improves the integrity of intestinal tight junction proteins via nod1/nf-κb p65 pathway in weaned piglets and h2o2-challenged ipec-j2 cells. J Funct Foods.

[bib88] Welk A., Otten N., Jensen M. (2023). Invited review: the effect of milk feeding practices on dairy calf behavior, health, and performance—a systematic review. J Dairy Sci.

[bib89] Xu J., Li X., Fan Q., Zhao S., Jiao T. (2025). Effects of yeast culture on lamb growth performance, rumen microbiota, and metabolites. Animals.

[bib90] Yu E., Chen D., Yu B., Huang Z., Mao X., Zheng P., Luo Y., Yin H., Yu J., Luo J. (2021). Manno-oligosaccharide attenuates inflammation and intestinal epithelium injury in weaned pigs upon enterotoxigenic *Escherichia coli* k88 challenge. Br J Nutr.

[bib91] Yu E., Chen D., Yu B., Luo Y., Zheng P., Yin H., Mao X., Huang Z., Yu J., Luo J. (2021). Amelioration of enterotoxigenic *Escherichia coli*-induced disruption of intestinal epithelium by manno-oligosaccharide in weaned pigs. J Funct Foods.

[bib92] Yu M., Meng T., He W., Huang H., Liu C., Fu X., He J., Yin Y., Xiao D. (2021). Dietary chito-oligosaccharides improve intestinal immunity via regulating microbiota and th17/treg balance-related immune signaling in piglets challenged by enterotoxigenic *E. Coli*. J Agric Food Chem.

[bib93] Zha A., Tu R., Qi M., Tan B., Liao P., Wu C., Yin Y. (2023). Mannan oligosaccharides selenium ameliorates intestinal mucosal barrier, and regulate intestinal microbiota to prevent enterotoxigenic *Escherichia coli*-induced diarrhea in weaned piglets. Ecotoxicol Environ Saf.

[bib94] Zhang Y., Tan P., Zhao Y., Ma X. (2022). Enterotoxigenic *Escherichia coli*: intestinal pathogenesis mechanisms and colonization resistance by gut microbiota. Gut Microbes.

[bib95] Zhao X., Wang X., Li H., Liu Y., Zheng Y., Li H., Zhang M., Cheng H., Xu J., Chen X. (2023). Comparative expression profiling reveals the regulatory effects of dietary mannan oligosaccharides on the intestinal immune response of juvenile megalobrama amblycephala against aeromonas hydrophila infection. Int J Mol Sci.

[bib96] Zhen W., Liu Y., Shao Y., Ma Y., Wu Y., Guo F., Abbas W., Guo Y., Wang Z. (2021). Yeast β-glucan altered intestinal microbiome and metabolome in older hens. Front Microbiol.

[bib97] Zheng L., Duarte M.E., Sevarolli Loftus A., Kim S.W. (2021). Intestinal health of pigs upon weaning: challenges and nutritional intervention. Front Vet Sci.

[bib98] Zheng Z., Huang Q., Kang Y., Liu Y., Luo W. (2021). Different molecular sizes and chain conformations of water-soluble yeast β-glucan fractions and their interactions with receptor dectin-1. Carbohydr Polym.

[bib99] Zhou Y., Luo Y., Yu B., Zheng P., Yu J., Huang Z., Mao X., Luo J., Yan H., He J. (2022). Effect of β-glucan supplementation on growth performance and intestinal epithelium functions in weaned pigs challenged by enterotoxigenic *Escherichia coli*. Antibiotics.

